# An Old Idea is a Novel Concept for Supplemental Funding of Surgical Residency Programs

**DOI:** 10.7759/cureus.7053

**Published:** 2020-02-20

**Authors:** Matthew Dimon, Bestoun Ahmed, Pam Pieper, Bracken Burns, Joseph J Tepas

**Affiliations:** 1 Surgery, Coosa Valley Medical Center, Sylacauga, USA; 2 General Surgery, University Of Pittsburgh, Pittsburgh, USA; 3 Pediatric Trauma, Wolfson Children's Hospital, Jacksonville, USA; 4 College of Nursing, University of Florida, Jacksonville, FL, USA; 5 Surgery, Quillen College of Medicine, East Tennessee State University, Johnson City, USA; 6 Pediatric Surgery, University of Florida College of Medicine, Jacksonville, USA

**Keywords:** surgical resident, value of resident participation, graduate medical education, current procedural terminology, medical education, supplemental funding of surgery residency

## Abstract

Background

In July 2014, the Institute of Medicine released a review of the governance of Graduate Medical Education (GME), concluding that changes to GME financing were needed to reward desired performance and to reshape the workforce to meet the nation’s needs. In light of the rapid emergence of alternative payment systems, we evaluated the financial value of resident participation in operative surgical care.

Methods

The Department of Surgery provided Current Procedural Terminology (CPT) codes for procedures performed by the general surgical service at our institution for the 2011 academic year. For each code, the charge and total instances were provided. CPTs allowing an assistant fee were identified using the Searchable Medicare Physician Fee Schedule. This approach enabled calculation of the potential resident contribution to GME funding.

Results

A total of 515 unique CPTs were potentially billable for a total of 6,578 procedures, of which 2,552 (39%) were reimbursable. These CPTs would have generated $1,882,854 in assistant charges. The top 50 most frequent CPTs resulted in 4,247 procedures. Within the top 50, 1362 procedures (32% of the top 50, 21% of the total) were reimbursable. Of the total assistant charges, $963,227 (51%) occurred in the top 50 most frequent CPTs.

Conclusions

Credit for resident participation in operative care as co-surgeon would average $67,244 per resident, compared to our current funding of $142,635 per resident. This type of alternative funding could provide 47% of current educational support. The skew in distribution of procedures also suggests that such a system could provide guidance to a more balanced operative experience. Such performance-based credentialing could be used to ensure appropriate housestaff for a given case; these reimbursements could also be adjusted based on quality metrics to provide for transformational change in patient outcomes.

## Introduction

Graduate Medical Education (GME) and its method of funding have changed in many ways over the past century. Prior to the 1940s, it was uncommon for new physicians to pursue formal GME programs. Most physicians simply began their practice directly out of medical school. In time, it became evident that advanced training was important for the development of clinical expertise and physicians began entering such programs in greater numbers. The cost of this additional training to society and the government was marginal because these physicians were only provided room and board, which was typically covered by the hospital endorsing the program. Such a system was aligned with both parties’ interests because it provided a source of cheap, skilled labor for the hospital while allowing for supervised clinical experience for the trainees. Further, the hospital was able to build the cost of trainees’ services into patient charges [1].

After World War II, there was an influx of veterans entering specialty residency programs. Over the next 20 years, the number of residency programs increased by 600%. This growth of postgraduate training was funded in part by federal support under the Servicemen’s Readjustment Act of 1944, which subsidized qualified resident training and provided a living allowance, as well as subsidizing hospitals that accepted veterans into their programs. This new approach to postgraduate training led to an increase in overall expenditure on GME due to increased numbers, as well as increased individual cost because residents began to expect more of a living wage during their time in training. This increased stipend was justified by them as providing support for direct “service” to the hospital and clinic in exchange for receiving higher education [1-3].

In 1965, Congress passed the Social Security Amendments of 1965, creating Medicare under Title XVIII of the Social Security Act as a social insurance program for individuals over 65 years. In doing so, Congress acknowledged the need to support medical education as well as direct patient care, stating that “educational activities enhance the quality of care in an institution, and it is intended, until the community undertakes to bear such education cost in some other way, which a part of the net cost of such activities (including stipends of trainees, as well as compensation of teachers and other costs) should be borne to an appropriate extent by the hospital insurance program” [4]. GME costs were authorized to be folded into the ”usual and customary cost” of providing patient care and were billed on a per patient basis. No limit was placed on the number of residents a hospital could train, and both private insurers and Medicare paid for per patient care based on these calculations [5].

In 1982, the Tax Equity and Fiscal Responsibility Act recognized the increased costs to hospitals associated with resident training. As such, limits on allowable hospital Medicare costs were increased based on a facility’s resident-to-bed ratio. Starting in 1984, to control rising costs, Medicare changed from a retrospective, cost-payment system to a prospective payment system based on Diagnosis Related Groups (DRGs). Under this system, hospitals were reimbursed for the actual cost of supporting residents via payments for Direct Medical Education (DME). The per-resident payment was determined by a hospital-negotiated, specific resident cost, adjusted for inflation, the number of FTE residents, and Medicare’s share of inpatient days for the facility. This payment was intended to cover resident salary and benefits, as well as supervising physician and support staff costs. Medicare also provided the so-called “indirect” payments for Indirect Medical Education (IME) to support hospitals in their teaching role and to cover added costs due to increased testing, greater severity of illness, the inefficiencies of time and expenses related to teaching, and the need to maintain cutting edge technology. This “indirect” reimbursement was initially set at a premium of 11.59% for each increment of 0.1 in resident-to-bed ratio. Over time, costs to the educational programs for this IME greatly overtook DME costs. In 1986, Medicare increased payments for hospitals treating a disproportionate number of indigent patients but decreased overall IME payments to 8.1% and then to 7.7% in 1989 [6].

In 1997, the Balanced Budget Act decreased IME payments further from 7.7% to 5.5% over 5 years. A cap was also placed on the total number of residents who could be funded through Medicare, as well as hospital resident-to-bed ratios. In 1999, the Balanced Budget Reconciliation act froze the IME payments at 6.5% [6].

In July 2014, the Institute of Medicine released a report on GME funding, which concluded that major changes were needed in the funding and governance of GME. They proposed a 10-year transition period in which the role of Medicare in GME funding could be altered [7]. If DME/IME costs are eliminated or even decreased significantly by this process, teaching hospitals will be forced to seek out other means to support their educational mission. Considering all these changes in light of the Affordable Care Act and its intent to transform the processes for reimbursement of medical care, we sought to determine the monetary value of a qualified senior resident functioning as a designated co-surgeon for certain operative procedures that are approved for such payment by the Centers for Medicare and Medicaid Services (CMS). As reimbursement continues to focus on value over volume of services and as the traditional model of fee-for-service is replaced by alternative payment methods, the true value of operative care as a component of disease management will require an accurate determination of resources required to provide an optimal outcome. A segment of those resources is in the contribution to care provided by appropriately qualified residents, which could prove to be an important component for the development of strategies for ongoing support of GME. 

## Materials and methods

The study received an exemption from the University of Florida at Jacksonville, College of Medicine Institutional Review Board. The Department of Surgery Business Group Manager provided an Excel spreadsheet file containing all Current Procedural Terminology (CPT) codes for procedures performed by the General/Acute Care surgical services with resident involvement at our institution between 1 July 2011 and 30 June 2012, regardless of payer. For each code, the charge and total instances were provided. CPT codes allowing an assistant fee were identified using the Searchable Medicare Physician Fee Schedule. This fee was calculated as 20% of the primary charge and multiplied by the number of CPT instances. This produced the total potential contribution by a resident for each code. These were summed to determine the total potential contribution to GME funding of the program by resident involvement (i.e. direct service to the care of the patient).

## Results

**Figure 1:**
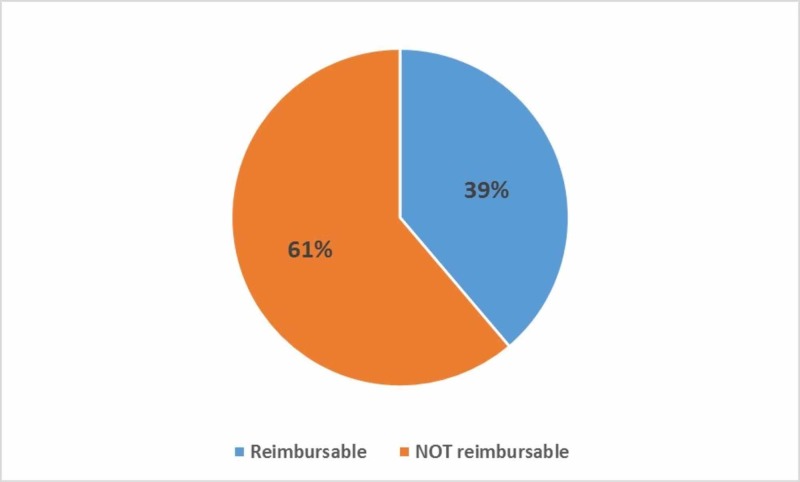
Percentage of procedures that were considered reimbursable for an assistant fee vs not reimbursable for an assistant fee per the Medicare fee schedule

**Figure 2:**
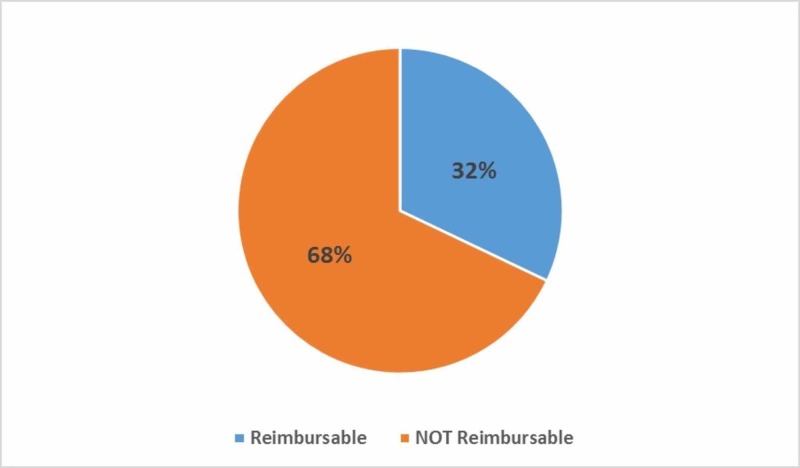
The top 50 most frequently performed CPT codes that were reimbursable for assistant fee vs not reimbursable for assistant fee per Medicare fee schedule

The caseloads of 15 attending surgeons comprising general and acute care surgical division faculties were audited. A total of 515 unique CPT codes were billed for a total of 6,578 procedures with direct resident involvement, of which 2,552 (39%) were designated as reimbursable for an assistant fee per the Medicare fee schedule (Figure 1). The top 50 most frequently performed CPT codes resulted in 4,247 procedures, representing 65% of the total. Within these top 50 CPT codes, 1362 procedures (32% of the top 50, 21% of the total) were reimbursable (Figure 2).

Allowable CPT codes would have generated $1,882,854 in billable assistant charges, in addition to the $18,570,909 in primary charges, for an additional 10.14% total revenue. Of the total billable assistant charges, $963,227 (51%) occurred in the top 50 most frequent procedures.

## Discussion

The federal government has borne a large share of the burden of funding GME medical training. Historically, Medicare has provided the majority of this funding ($9.7 billion in 2012), supplemented by the Department of Veterans Affairs, which subsidizes approximately 10% of residency positions ($1.437 billion). Other contributors include the Department of Defense and Medicaid ($3.9 billion) through the policies specific to several states [7]. The American public benefits from this expenditure by using the availability of a highly trained and competent physician workforce. Additionally, teaching hospitals serve an important role in the provision of care to indigent, underserved, and marginalized populations, which otherwise may be neglected. Teaching hospitals also serve as breeding grounds for scientific inquiry and advancement of medical science, which also benefits the population as a whole, albeit indirectly, through the development of new therapies, techniques, and procedures for treating disease. Despite this overall benefit of GME to the American public, there has been considerable discussion and controversy with respect to the future role of Medicare funding of GME, particularly given the concern about the long-term solvency of the Medicare Trust Fund. Even if the primary purpose of funding physician training is not considered a public good, the byproducts of these programs, such as indigent care and medical research, certainly are direct benefits to our populace [8].

Over the years as teaching hospitals’ ability to cost-shift has decreased, there has been increasing reliance on the GME funds from Medicare to support resident training directly, as well as to subsidize often unfunded missions of safety-net care and research [1,7-8]. With increasing emphasis on the uncontrolled upward spiral of cost of healthcare, there has been much pressure to decrease the role of the federal government in funding medical education. It has been argued that the direct costs of medical education are inappropriate for public funding because residents are in essence incompletely trained physicians who pay for their education with their labor. In the traditional fee-for-service environment, provision of direct fee payment to co-surgeon residents for participation in a process that is a part of training could be perceived as a transformation of student to provider. As alternative payment systems mandate a more accurate assessment of resources necessary to provide optimal care for various bundled services, understanding the cost contribution of resident participation (i.e. “service”) in the provision of this care will be essential to determine fair reimbursement. In fact, emerging priorities in surgical education mandate more emphasis on consideration of resident contribution to the evolving team-based surgical work product. Currently, the cost of resident operative assistance is an undefined part of IME. Our data define the value of this contribution more precisely and could be used to justify and link allocation of educational support to surgical training. As a defined component of the cost of operative care, it would also spread the process of GME support to all payers.

In 2012 the Institute of Medicine was charged with reviewing the governance and financing of the GME system. The investigators concluded that while Medicare has, over the past 50 years, funded a system that has progressed to a robust, curriculum-based program with improved resident working conditions and decreased gender and racial disparities, the systems of GME have failed to keep up with many changes in modern medicine, including the increasing role of non-hospital-based care in the management of chronic medical conditions. As it stands, GME payments are essentially guaranteed because they are based on legislated formulae. The report of the Institute of Medicine suggested that the ongoing investment into GME by Medicare be leveraged to redesign the system to reward desired outcomes and performance of training programs to produce a workforce that fits today’s needs while remaining budget-neutral. This transformation would likely include the redistribution of substantial portions of the budget from hospital-based training to ambulatory and community-based training, as well as shifting money from specialty training to primary care programs [7]. Thus, it is imperative that the value of resident contribution (i.e. “service”) in providing inpatient and acute care be defined and factored into guidelines for the appropriation of GME support.

In the recent past, both houses of Congress and the Administration have recommended decreases in GME funding. As the Affordable Care Act continues to evolve, its intended goals of increased coverage, better quality, and less cost will continue to drive a dramatic transformation in processes and policies for reimbursement. The intent of the recently passed PL114-10, the Medicare Access and CHIP Reauthorization Act (MACRA), is to replace the traditional system of fee-for-service payment with alternatives that pay for disease management or, possibly, procedure-based global care. This “bundling” will reward effective and cost-efficient care, as well as penalize institutions and providers whose performance is below expected. A major part of understanding this approach is the determination of what it really costs in terms of personnel and resources to provide optimal care for every surgical patient, especially for complex procedures where experience and teamwork are paramount. It makes sense, therefore, to “cost out” contribution to this care of appropriately-credentialed residents as designated co-surgeons and, in fact, to include this contribution (i.e. direct “service”) as part of the anticipated cost of care.

Analysis of one year’s billing for our general surgery service suggests that using the co-surgeon fee as a surrogate can define a substantial and justified monetary contribution by senior residents to the delivery of clinical care. Focusing only on cases that would qualify for an assistant surgeon under current guidelines, the identified revenue is both part of what is necessary for the delivery of optimal care as well as an offset for the cost of educating future providers of the same care. If appropriate, co-surgeon operative experience was distributed evenly within the program, the equivalent of $67,245 would be generated per resident per year. In comparison, under the current Medicare formula our facility receives $82,386 in DME and $60,249 in IME per resident per year. While this approach would not fully fund resident education at its current levels, it would link almost half of program costs to resident participation (i.e., “service”) in delivery of operative care and define the contribution of their labor to the totality of services provided. Because private insurers are also aggressively pursuing alternative payment strategies based on procedure or diagnosis bundling, this system would more equitably distribute some of the educational support to this sector.

An essential component of an alternative payment system that accounts accurately for resident contribution is a competency-based process under which resident surgeons, as they progress through their training, become certified to provide the level of support services that are part of the cost of care. Medicare and many private insurers currently allow certain procedures to be performed with the assistance of a second surgeon who is reimbursed at approximately 20% of the primary surgeon’s rate. Because teaching hospitals typically utilize residents as assistants to provide this “service” in such cases, this contribution to care by the resident could be included in calculations that define adequate reimbursement for such services and could be cost-accounted to educational support. Furthermore, a system could be designed to adjust co-surgeon reimbursement via a multiplier based on a hospital’s reported outcomes on key educational measures, providing a further financial motivation to drive desired changes in the performance of training programs. Such measures of outcome become an important consideration as reimbursement shifts from fee-for-service to alternate payment methods driven by value. 

The traditional surgical residency as first proposed by Halsted was based on full immersion of the trainee in the operative technique and comprehensive care of surgical in patients. The equation of scholarship versus service has always mandated primacy of the former but, as the process of valuation of surgical care continues to change, it is critical for us as educators to justify reimbursement for resident “service” (and education). The new paradigm for resident training emphasizes performance as a part of an integrated team whose collaborative effort yields better care and greater patient satisfaction at less cost. Recognizing that resident effort as part of training in this new process also adds to the consumption of resources associated with the direct care of a particular disease or an otherwise bundled procedure, we should begin to factor in this “service work” provided by our residents to assure that the reimbursement system is, in fact, able to support GME [9].

Surgical trainees are not and must never be considered as revenue-generating workers. Their “service” in the actual provision of care, however, must be accurately cost-accounted to assure that reimbursement is equitable and to define a potential revenue source to support their training. More comprehensive governance of residency training programs, which was another recommendation of the report from the Institute of Medicine, will be essential to assure accurate accounting of service-related components of residents’ labors while optimizing the quality of their ultimate training.

## Conclusions

Direct provision of healthcare remains a core characteristic of GME; however, determination of the value of this “service” and assuring that it is recognized appropriately as a component of clinical care may prove to be essential for the survival of our nation’s commitment to the medical care of its citizens. While any solution to the proposed decreased GME funding will be multifactorial, our findings regarding the value of the resident contribution to operative care could play a key role in the design of systems for continued funding of training programs, as well as help to reshape them to meet the needs of society now and into the future. Obviously, such an approach would require buy-in by Congress as well as other policy makers, and we as educators must be prepared to assist in this potential transformation of the culture of surgical education.
